# Barriers to Accessing Addiction Treatment for Women at Risk of Homelessness

**DOI:** 10.3389/fgwh.2022.795532

**Published:** 2022-02-17

**Authors:** Davinia Rizzo, Temika Mu, Sophia Cotroneo, Shalini Arunogiri

**Affiliations:** ^1^Faculty of Medicine, Nursing and Health Sciences, Monash Addiction Research Centre and Eastern Health Clinical School, Monash University, Richmond, VIC, Australia; ^2^Turning Point, Eastern Health, Richmond, VIC, Australia

**Keywords:** addiction, treatment access, women, lived experience, homelessness

## Abstract

Women remain under-represented in addiction treatment, comprising less than a third of clients in treatment services. Shame, stigma, and fear of legal and social repercussions (e.g., child protection involvement) are major barriers impacting on treatment-seeking for women. This is compounded for women at risk of homelessness, with practical and logistical reasons for not engaging in treatment. We conducted a qualitative study with both clinicians and service-providers, and women with lived experience of addiction and at risk of homelessness, to identify barriers to access and help-seeking within this vulnerable population. Adult women with lived experience of homelessness and addiction were invited to participate in an online focus group. Interviews were transcribed and analyzed using framework analysis. Analysis resulted in the identification of barriers to access in three areas. These were system-related, socio-cultural, and emotional barriers. We also present findings from the focus group recorded in real-time, using the novel method of digital illustration. This study highlights key factors impacting on help-seeking and access to treatment for addiction faced by women at risk of homelessness. The findings of this study highlight important areas of consideration for clinicians and service-providers working with women who experience addiction, as well as informing future research directions for this priority population. Findings are discussed in the context of exigent literature.

## Background

The relationship between alcohol and other drug (AOD) use and homelessness is complex. An individual can be defined as homeless if their current dwelling is inadequate, has no tenure, or tenure is short and not extendable, or the dwelling does not allow an individual to have control of, and access to space for social relations (1). There is no clear evidence for a causal link between the two however there is clear evidence that AOD use, and abuse is a significant issue for people who experience homelessness (2–4). Upshur et al. surveyed women across nine health care for homeless clinics in the United States (5). They concluded that when compared to the general population of women, homeless women were four times more likely to be diagnosed with alcohol use disorder and 12 more likely to be diagnosed with a drug use disorder. In Australia, the Australian Institute of Health and Welfare has collected data exploring the intersection of alcohol and other drug (AOD) use and experiences of homelessness over several years (6). In 2019-2020, 1 in 10 clients of Specialist Housing Services (SHS) reported problematic AOD use (6). Clients had significantly more health issues, were homeless for longer periods, drew upon supports for longer periods of time and were more likely to be homeless at the end of a support period than SHS clients without problematic AOD use (6), highlighting the complex and chronic nature of substance use and homelessness in Australia. Limited access to treatment services, experiences of stigma, person and service level barriers are regularly reported in the literature as barriers to addiction treatment specifically (3, 7), and health care more generally (8, 9) by individuals who are experiencing homelessness.

Pathways to homelessness are wide and varied. From an interactionist perspective, homelessness can be understood as a contextual situation, underscored by a complex combination of human agency and structural factors, including, but not limited to poverty, financial stress, inadequate and unaffordable housing, mental health and/or substance use (10, 11). Pathways to and experiences of homelessness can differ between men and women (12–14). In addition to the aforementioned underlying causes, factors which could affect a women's homeless status included their own chronic (mental or physical) health conditions (15–17), or their experiences in caring for another with chronic health conditions, harmful and /or violent relationships (18), and substance use issues (19). Women who are homeless report high rates of historical trauma (20, 21). Once homeless, women continue to experience high rates of assault (22) and victimization (23). Homelessness can also adversely impact upon pregnancy and the experience of motherhood. For example, having unstable accommodation can hinder access to reliable contraception and pre-natal health services (24, 25). Furthermore, navigating motherhood in the context of transition houses or shelters raises specific issues in respect to parent-child relationships (26).

Experiences of AOD use and use disorder also differ across gender (27–29). Not only does the etiology vary (29, 30), but research continues to confirm the differential experiences of AOD use generally and substance use disorder (SUD) specifically across the sexes (31, 32). Experiences of treatment and recovery from SUD can also differ across gender (33, 34). Pregnancy, childbirth, and motherhood are regularly reported as factors preventing women from accessing AOD treatment (35–38). Women are also at an elevated risk of experiencing co-occurring mental health or personality disorders in comparison to men (39–42). Furthermore, shame, stigma, and fear of legal, and social repercussions (e.g., child protection involvement) have also been identified as barriers which can impact upon women seeking AOD treatment (38).

Despite the identification of such barriers, the number of women accessing AOD treatment remains low, and overall, women are underrepresented in treatment. In Australia women made up only one third of recorded public treatment episodes for SUD in 2019-2020 (43). Of those seeking support from Specialist Housing Services (SHS) in Australia, 60% are female (6), in 2019-2020 this equated to ~170,000 clients. Yet, despite the inherent links with problematic AOD use, reports indicate that only 0.8% of women seeking SHS support were referred to AOD treatment services, and only 0.4% or 728 women were provided with AOD counseling. Furthermore, recent economic modeling suggests that even in the presence of a universal healthcare system, out-of-pocket expenses pertaining to health care expenditure inexorably effect lower income earners (44). Taken together, these circumstances suggest that women who experience co-occurring AOD use, and homelessness are at an extraordinarily increased likelihood of not being linked into treatment, compared even with women who use substances but who do not access SHS.

Experiences of AOD use and homelessness interact bidirectionally to prevent women from accessing and engaging effectively in treatment, perpetuating the cycle of disadvantage, and hindering efforts of recovery (5, 45). Despite recognition of the barriers faced by women living with homelessness and SUD, there remains little research into this experience from the perspective of those who live it. Kneck et al.'s study exploring women who experienced homelessness engagement with health services in Sweden is one exception (46). They identified three themes which underscored these women's capacity to access services. Firstly, a demand for a life in order, considered the conditional nature of health care access for this cohort and the requirement for women to have a suitably stable lifestyle. Secondly, the theme of being unwell, unsafe and a woman, explored the multifaceted needs of women as a challenge to the health care system. And finally, the theme of abuse vs. humanity, spoke of the power within health care encounters to reduce or elevate the patient. Despite the importance of these findings, it remains that an exploration of the experiences of women who are homeless and their capacity to access AOD treatment services has not been undertaken.

The aim of this study was to address this gap in lived experience perspectives on the barriers to AOD treatment faced by women with addiction and homelessness.

## Method

This study was a qualitative research project undertaken at a specialist public Australian addiction treatment center, Turning Point, as part of a broader service development initiative aiming to address women's needs in care. This project was approved by the Monash University Human Research Ethics Committee (MUHREC # 24562).

Inclusion criteria for the qualitative study comprised adult women at risk of homelessness (currently homeless or engaging with SHS due to their risk of homelessness), who also had a history of substance use disorder and a history of seeking treatment for this. Women were recruited through the service's networks and by approaching service providers and clinicians. Potential participants were asked to speak with the research staff directly to express their interest in participating. Following receipt of an expression of interest, research staff provided potential participants with relevant information and documentation. This process was facilitated by the rapport developed between housing service staff and participants and included addressing practical issues such as access to telephones and internet.

Women were invited to participate in a focus group, which was held online (by Zoom) due to COVID-19 related lockdown restrictions. Participants were reimbursed AU$50 (in voucher form) for their participation in the focus group. For women who had challenges accessing suitable technology, they were invited to attend the clinical service on-site and were supported to attend the focus group from a clinical room computer.

The focus group was completed in October 2020. The focus group ran for approximately 90 min and was led by SA, with support from DR and TM. Seven women were recruited; all participants were over the age of 18 years and had experience with addiction treatment services. They, were, or were at risk of becoming, homeless. Three women attended the clinical service on-site, two women were in residential rehabilitation units [accessing devices provided by the service (i.e., tablet)] and two were in private accommodation; using their own devices.

The focus group discussion explored the following questions,

- How do you access addiction services?
- What are the barriers to accessing these services in Victoria?
- How do these barriers affect your capacity to access these same treatment services?
- How do you think services could be adapted/altered or changed to address the identified barriers?
- How/when do you provide feedback services?
- What are the strengths and limitations of these feedback processes?
- What would you like to see in a feedback program, how would you access and use this?

The focus group was recorded via two processes simultaneously. The first method involved graphic recording, with a professional live scribing artist providing the development of an *insitu* output; a digital illustration. The document provided a novel way of reporting to stakeholders the findings of the focus group, as well as providing real-time reflection of the key issues being raised in discussion. The artist employed had prior experience working in the context of vulnerable populations and in the addiction space. The artist completed a confidentiality form, attended the focus group, and proceeded to visually summarize the information collected *via* a digital platform. The second, more traditional method, was the audio-visual recording of the focus group. This was captured, with participants consent, *via* Zoom functionality. The recording was transcribed verbatim by DR and reviewed by TM and SC for accuracy.

As a study in implementation, framework analysis was used as the primary methodology (47). Developed in the context of healthcare services, with the aim of identifying barriers to services, analysis is undertaken at two levels. Deductive analysis provides for the analysis in the context of a specific question or questions. In the current context, deductive analysis allowed for the exploration of the barriers specific to women at risk of homelessness accessing substance use treatment. Inductive, or “bottom up” analysis aims to identify the thoughts and feelings of the participant regarding the context under consideration. This dual process provided for the identification of practical barriers to treatment, and a deeper exploration of the lived experience of the participants. It is this specific knowledge that can facilitate services to address the barriers in a way that is consumer focused and inclusive.

Analysis occurred iteratively, in which the authors' experiences of recruitment, planning and implementation of the focus group, the transcript and the digital recording were reviewed both independently and later, simultaneously. For example, coding of the transcript was undertaken, and initial themes were then examined in the context of the digital illustrations and the lived experience of the focus group. This process occurred over several months to provide significant reflection and discussion between authors. Final themes were developed by DR and confirmed by SC and TM.

## Findings

### Digital Scribe

**Figure 1:**
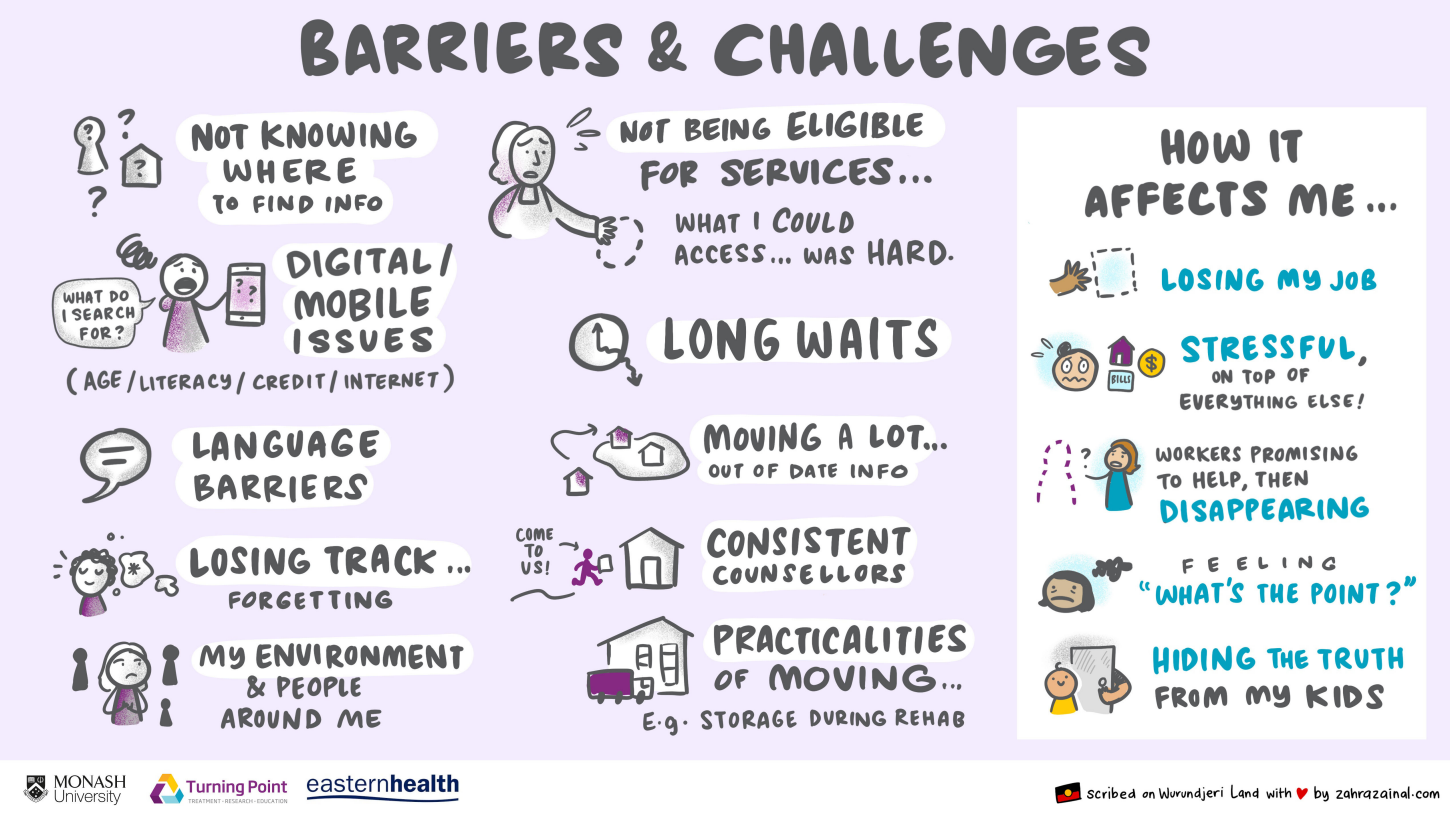
Barriers and challenges.

**Figure 2:**
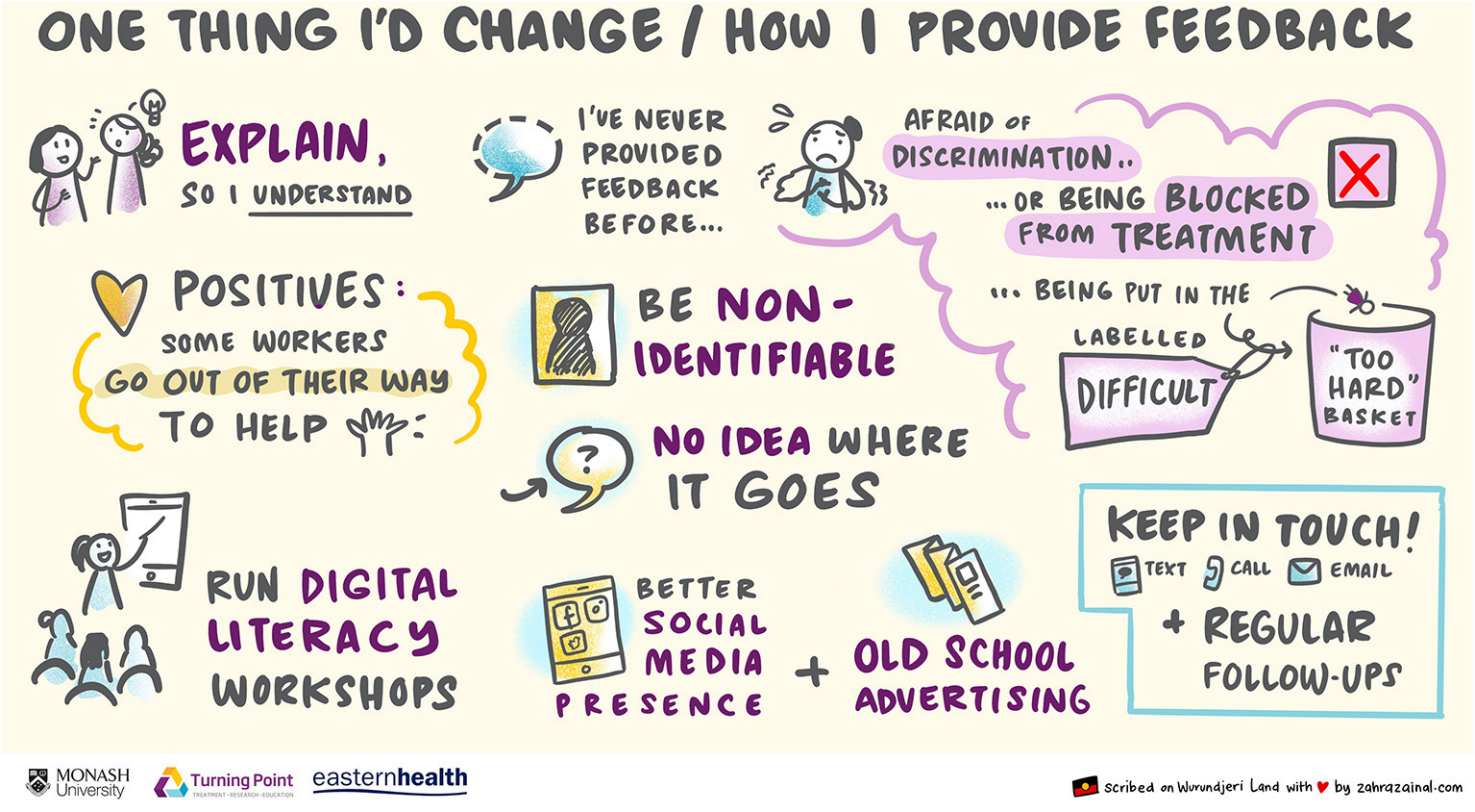
One thing I would change/how I provide feedback.

The digital scribe completed the recording of the focus group in real time, the images below (Figures 1, 2) were derived using the language and experiences of the participants directly. The incorporation of the graphic recording allowed for real-time reflection, within the focus group, of the themes that were raised. Both during, and at the conclusion of the discussion, participants were asked to review the images and provide feedback as to their accuracy.

### Framework Analysis

Deductive analysis of the focus group transcript sought to authenticate and develop further the results of the digital scribe. As such, analysis identified the barriers experienced by participants when accessing treatment services. Two themes were identified during this analysis, system-related, and socio-cultural barriers faced by the women in their efforts to access treatment. Inductive analysis provided a space in which we could explore participants' responses to the identified barriers and how they made sense of their experiences with treatment services. In doing so we were able to explore more nuanced barriers to addiction treatment. This second level of analysis resulted in identification of the emotional barriers to treatment. These responses mapped directly onto the themes identified in the deductive analysis.

#### System (Related) Barriers

The women identified several immediate barriers pertaining to accommodation stability, which had, at one time or another, limited their access to AOD treatment services. Participants reported that under a catchment-based allocation of treatment services, having no fixed address meant they could not be waitlisted for a specific service. In the case of frequent changes of address particularly across catchments, eligibility for services was often jeopardized. In these cases, once a change of address was identified, the participant's place on any single waitlist would be lost. As one participant noted,

“*…you don't know what suburb you are going to be in, do you know what I mean, it is like you are pretty much like wherever you can be and then someone won't take you because you are not like within that zone or whatever”*.

The women also spoke of the impact that chronic homelessness had on their capacity to attend different addiction treatment services. Attendance at long-term residential rehabilitation was not conducive to receiving or maintaining a property in the community. Outpatient treatment was contingent on all other aspects of their circumstances- housing, transport, finances-to be sufficiently secure to permit them to attend an appointment at a specific time and place. Just staying in contact with services could be difficult,

“*…not updating your phone number and address, so you just sort of get, yeah, they are obviously trying to contact your old place and you know, your three new places ahead or something….”*.

“*Someone will steal your phone, or your handbag is gone… you try to reconnect with whoever you made the appointment with, and it is too late”*.

Managing eligibility criteria, holding a place on wait list and maintaining contact with services were all impacted by issues related to housing instability. Once admitted to treatment, participants had to navigate appointments and maintain contact with services all within the context of their socio-cultural barriers.

#### Socio-Cultural Barriers

Social and cultural barriers to addiction treatment included multiple levels of illiteracy, socially constructed gender roles, and the chaotic nature of participants' lifestyles. Barriers were further reinforced in the face of treatment staff limited understanding of their circumstances.

As a group, participants reported low levels of literacy. In the traditional sense, literacy was expressed in terms of education. However, literacy deficits were further affected by ongoing substance use. Both directly, for example ongoing cognitive difficulties in comprehension and memory because of regular and sustained use, and indirectly, for example one participant reported the impact of an Acquired Brain Injury on her literacy; received when, under the influence of substance, she had a car accident. Participants reported that sometimes they just did not understand the language being used by clinicians or service providers. As this woman noted,

“*If there was something that I didn't understand, I would put it to their attention. I would say listen, I can't understand, can you please put it in a different context where I can understand”*

In the instance that traditional literacy was sufficient to access service information, participants reported that they just did not know where or how to obtain information that could assist in their accessing treatment. Health literacy was poor, participants were unaware of services and treatment types available.

“*…not knowing who to go to for what and just trying to sort of find out yeah, what is out there”*

Participants felt that they had been adversely affected by the transfer of information to digital platforms. Digital literacy was low and access to devices like smart phones (and associated consumables) was limited by the women's earning capacity and the insecurity of homelessness.

“*Even when I did have a phone cos I am not like very good with computers and stuff, like I would not have had a clue, like I wouldn't have a clue how to access anything”*

“*… can't afford data…. or credit, or whatever yeah you are sort of stumped.”*

Low levels of literacy, across multiple domains, limited participants' ability to access treatment, both in respect to traditional conceptualization, but also in respect to health services and the use of digital devices. Taken together these made it difficult to access the information available, and then to understand what this meant for them specifically.

The women also highlighted the limited suitability of specific programs in respect to their lives. Further to these service level barriers, traditional gender roles inhabited by these women meant that as mothers and carers they had to deal with the day to day needs of their children and other dependants. Inpatient treatment facilities and long-term residential rehabilitation were not viable options in the instance that the women were primary caregivers. Whilst outpatient services continued to operate on a traditional 9-5 structure and provided limited flexibility in appointments. As one woman noted,

“*if you miss an appointment like to be assessed for detox or something…you have to start all over again”*

Housing, feeding, clothing, and managing the care of the family became a top priority, their own health and wellbeing—including issues of addiction were “the bottom of the list.”

“*…everything else being so hectic you tend to yeah, it [your own health and wellbeing] just gets push aside…”*

Building upon this list of demands is the biology of addiction itself,

“*…you wake up and that is what you think about doing, and then till you get it…you can't function …. everything else stops, and then once you have it you can sort of function… but then the day is nearly over, and appointments are gone”*

This statement also alludes to the chaos which characterizes the lives of these women. Neatly summarized by one interaction between two participants, who continue,

“*P6: Just also the distress of all of it, like when you are oh great, I have an appointment with ____ at housing or whatever and you will go there and it won't happen…the stress of all that, not getting a result when you are trying, you know what I mean?*

*P5: Or you get disheartened, yeah*,

*P6: especially when you have other people to look after and things like that, it gets a lot, you know*

*P5: … like and with the anxiety you are getting, like trying to keep appointments, and keep the phone and keep the family and the house all operating at the same time so you won't be homeless…as were about to be…you know what I mean, often that gets, it just gets so much…even getting to this, like even getting to this today was a major event, Okay”*

For the women we interviewed managing the practical and emotional aspects of their day-to-day experiences was overwhelming and exhausting. In this context, there is little space to make treatment a priority.

Whilst the socio-cultural milieu of the women who exist in this space between addiction and homelessness not only thwarted efforts to access treatment, during the conversation, it became clear the women believed that generally, service providers did not understand their lives. This was noted by the staff that stood out to the participants,

“*____ has been fantastic, but I mean right from the start she knows how hectic and crazy our life can be and she gets [sic] and please can you send me the details the week before, so I don't stuff it up and all that sort of stuff.”*

And in comments such as,

“*I know that people like you guys do the best you can”*

Whilst acknowledging the support she received from staff in respect to the issue at hand (receiving assistance with computer access), underlying the initial sentiment is a belief that treatment staff cannot really understand the circumstances these women face.

The women interviewed painted a picture of a life that is restricted by multiple socio-cultural barriers. They are unfamiliar with the services available to them and how to access these. They experience discrimination in the face of a digital society, either having limited access to, or being illiterate in the use of such this technology. Their roles as mothers are not realized by the services they seek to access. Their efforts to manage these obstacles in the context of active addiction and homelessness are further limited by service provides who are naïve to the realities of their lifestyle. When faced with the multiple complexities of their circumstances, participants felt that services put them in the “*too hard basket*^1^*.”* Another felt that the socio-cultural barriers left services saying,

“*nah, that is too hard, not dealing with this”*

### Emotional Barriers

The emotional experiences of the women interviewed in response to the system-related and social-cultural barriers navigated removed them further from the treatments they sought to access.

Participants expressed frustration with a system that did not sufficiently meet their needs. Eligibility criteria and long waitlists were compounded by their unstable accommodation. They felt that services disregarded their roles as mothers and carers and that they failed to understand the sociocultural circumstances they inhabited. A comment which was noted in respect to the effect of high staff turnover, but nonetheless reflected participants wider experiences,

“*yeah well sometimes you will be in contact with one person and you will think great this person is really helping me and then they will just disappear and you won't have that…um I'm just making up a name…Sally….but Sally from such and such is telling me she is going to do this and then she is gone and you just kind of get lost in the system again”*

As mothers these women also had to deal with the all too real implications of reports from treatment services to family services regarding both their accommodation issues and their substance use. They expressed an increased level of anxiety when discussing the feelings of vulnerability they encountered when accessing services.

“*…particularly when you have children, you want to hide your problems, because DHHS*^2^*, and all the rest of it…and then you are worried about losing your children too”*

But it was not just the mothers who felt vulnerable. As a young female, one participant explained the feelings of increased vulnerability in the detox space.

“*…they sit you in _____ with addicts that are a lot older than me, I am only 24, and I was sitting in there with you know, with older people who were much heavier addicts, who were sitting there asking for Valium, Ahhhh, just yeah, it was pretty horrible, I won't lie”*

Finally, the women had to manage the stigmatization they felt when accessing services, they reported embarrassment, shame and distress associated with their lives and substance use.

“*P5: you try and think that you are in control of everything and yeah you just don't want to, I suppose, being honest and open, you know you feel like you are going to be, I don't know, criticized or stereotyped…*

*P6:…it's embarrassing….”*

Frustrated, and vulnerable, they felt abandoned by the system that was advertised to support and assist them.

“*they are just druggo's do you know what I mean, don't give a shit, you know it feels like that sometimes”*

Attempts to access and engage in treatment, increasing frustration, and emotional distress enhanced vulnerabilities, and reinforced stigma in the lives of these women, resulting in entrenched feelings of hopelessness and helplessness. The emotional experiences of these women not only reduced any capacity to engage in the process of treatment but in many cases resulted in the opposite—a return to substance use,

“*You think kind of like ‘fuck it' like why bother and like___ said, you go and get high again”*

“*You just go what you have been doing [using substances] because they are not going to help, you know it becomes a comfort, so you know it becomes easier I think”*

## Discussion

The wider literature suggests women access services at reduced levels compared to their male counterparts and those who experience homelessness are further restricted in their capacity to access treatment services. To explore the intersection of gender and homelessness as a barrier to addiction treatment, we recruited seven women with lived experience of addiction treatment who were either homeless or at risk of homelessness to participate in a focus group. Our findings are presented in two parts, the output of the digital scribe and the themes identified via framework analysis. Whilst these were separated for presentation, it is of note that our discussion is reflective of the interconnection between the practical barriers identified, the emotional experiences of the women involved and the experiences of the focus group itself. Our findings support the small but growing body of research exploring women's access to AOD treatment in the context of homelessness.

Our findings are consistent with the limited extant literature. In one of the few studies identified exploring barriers to AOD treatment experienced by this cohort, women attending homeless healthcare services across nine US sites were surveyed in respect to the type of AOD treatment they had accessed and the barriers they experienced when accessing this treatment (5). Women with three different experiences of addiction were surveyed, those who reported alcohol use only, those who reported drug use only and those who reported use of both drugs and alcohol. The authors identified barriers to treatment from the relevant literature and asked women to identify which they had experienced. Barriers heavily endorsed by those surveyed were reflected in the current findings, including limited program availability, low levels of health literacy and extended wait times for services to become available. Across all three groups of women, the most frequently endorsed barriers were “feeling depressed/not up to going to treatment” and “too busy.” While methodological differences preclude any direct comparison between studies, our findings may provide insight into these highly endorsed barriers. The women we spoke to felt frustrated with and ignored by AOD treatment services. In the context of these experiences, it is not unexpected that motivation to attend AOD treatment services is reduced. This could also reflect a practical issue in which they do not want to, or are unable to, manage the process of attending AOD treatment services in the context of ongoing substance use and homelessness reported by some of our participants. In these instances, participants felt they were at the “bottom” of the to-do list; this may potentially reflect the circumstances of other women in similar situation being “too busy” to attend treatment.

We highlight these comments for discussion, for as clinical staff, it is not uncommon to hear “I am too busy,” or to have the experience of not wanting to engage with an “unmotivated” client. However, taking statements like these at face value perpetuate cultural misunderstanding and reinforce the discrimination these women experience. The women we interviewed were keen to have the research team understand the chaotic nature of their circumstances and the barrier(s) this raised when attempting to access treatment services. Like an entangled ball of twine, to pull one thread, just causes another knot to tighten. A common scenario amongst the women we spoke with is laid out as an example. Their roles as mothers and carers meant that inpatient or residential options were not practical. They may receive an opportunity to engage in outpatient sessions, however this can be reliant on their locale and the need for a fixed address to be waitlisted. In the instance that they secured an appointment, they had to be able to record this securely, attend this appointment—finances permitting—on time, with all relevant documentation. They may have to attend this appointment with children, or alternatively, rely on family or friends to provide free childcare.

The existing structure of treatment systems demand “a life in order,” and this is unrealistic for women living with homelessness. Intersectional stigma posits that individuals may experience stigma resulting from the dynamic interaction of multiple marginalized social identities (48, 49). Recently, intersectional stigma has been used to explore the experiences of women in this space (50). Our findings build upon this work by providing specific examples of how intersecting stigmas can present themselves as barriers to treatment. In one example a participant describes feeling shame and embarrassment regarding her circumstances (internalized stigma). She reflects that between her own feelings and the belief (anticipated stigma) that family services would be employed (enacted stigma) should she engage with services, her motivation to access treatment services was reduced. Development of a comprehensive understanding of the sociocultural context of women managing both homelessness and addiction will be an important step in developing treatment services that facilitate, not hinder access.

Knowledge of systems and services provides people with the capacity to make informed choices about their own circumstances, this includes when, where and how to access treatment and health services. Poor literacy and socio-cultural context intersect with addiction to prohibit this group of women from being active participants in their own treatment and recovery. One participant noted that sometimes she just did not understand the language that was used by services and clinicians, another noted that she did not even know “what to search for” when she had wanted to engage with treatment services. Another lamented the wholesale move of information and services, for example, counseling services and peer-based recovery groups, onto digital platforms (which was only expedited in the context of the COVID-19 Pandemic), noting not only limited capacity to navigate digital platforms but limited access to technology.

A lack of information or the capacity to access information, not only left the women interviewed feeling helpless and hopeless in their efforts to access treatment services, low levels of literacy, traditionally, and in respect to health and digital technology, has rendered these women almost powerless. Research highlights the importance of self-efficacy in achieving and sustaining good health, both physically and mentally (51, 52). In the context of poor literacy and the limitations of their sociocultural circumstances they felt shamed, embarrassed, and stigmatized, none of which provide for the growth of self-efficacy, a vital component of successful addiction treatment (53–55).

Taken together similar findings across time and place, suggest barriers preventing women who experience homelessness from accessing AOD treatment involve a complex interaction of individual, service, and wider social characteristics. Due to the limited research in this specific context, strategies of potential redress must be sequestered by exploring the wider literature. Exigent research explore the importance of developing a genuine understanding of individuals' complex lives (25, 56), as well as the importance of prioritizing good relationships between staff and those using services (7, 56, 57). The women we spoke with reported feeling uncomfortable exploring their circumstances with services providers none the less, they spoke openly about their experiences during our focus group. This discrepancy suggests that when provided with a space in which they do not experience fear of punitive state systems (child protection or legal systems) or ongoing stigmatization, they feel comfortable discussing their experiences and the potential issues they are facing.

Our experiences with the focus group also reflect suggestions that a person-centered approach to care (56, 58) and the engagement of the user in treatment services at every level is crucial. Participants reported that they had never given feedback to the services they accessed (see Figure 2), engaging the experiences of this group can provide services with a better understanding of their experiences and circumstances, but also provide these women with an increased opportunity and competency to articulate their experiences.

Using traditional and novel methods of data collection we were able to contribute to a comprehensive understanding of women's experience. The capacity for women in this context to access AOD treatment services is limited by systemic barriers, but also due to individual factors, for example low levels of digital and health literacy and socioeconomic disadvantage. Emotional barriers to treatment access were also identified, and their overlap with the individual and systemic barriers identified supports the use of intersectional stigma as a framework from which future research into this cohort can be undertaken.

In conclusion, growth of this body of literature provides practitioners with an understanding of women's experiences and, importantly, scope to addressing these issues. The current study was undertaken with the aim to explore the lived experience perspectives as to the barriers to AOD treatment faced by women with addiction and homelessness. This study contributes to the expanding body of lived experience perspective literature and highlights the value of multi-dimensional perspectives in informing service delivery. Whilst the women we spoke with identified multiple barriers to accessing AOD treatment, when efforts were made to address these issues, for example supporting access to technology, they were motivated to engage, and open up about their experiences. Incorporating lived experience perspectives into service development offers an opportunity to design services that take into account the real-world barriers that exist, and to adapt interventions using a gendered lens to support more effective engagement.

## Data Availability Statement

The raw data supporting the conclusions of this article will be made available by the authors, without undue reservation.

## Ethics Statement

The studies involving human participants were reviewed and approved by Monash University Human Research Ethics Committee. The patients/participants provided their written informed consent to participate in this study.

## Author Contributions

SA conceptualized and designed the research project. SA, DR, and TM undertook data collection. DR, TM, and SC analyzed the data. DR wrote the first draft of the manuscript. All authors contributed to manuscript revision, read, and approved the submitted version.

## Funding

The research project was funded in part by the Victorian Women's Benevolent Trust, this funding body had no influence over the research conducted or the publication process.

## Conflict of Interest

The authors declare that the research was conducted in the absence of any commercial or financial relationships that could be construed as a potential conflict of interest. The handling editor declared a shared affiliation with several of the authors DR and SA at the time of review.

## Publisher's Note

All claims expressed in this article are solely those of the authors and do not necessarily represent those of their affiliated organizations, or those of the publisher, the editors and the reviewers. Any product that may be evaluated in this article, or claim that may be made by its manufacturer, is not guaranteed or endorsed by the publisher.

## Footnotes

^1^Participant 7.

^2^Department of Health and Human Services is the state service responsible for child welfare in Victoria Australia.

## References

[1] Statistics ABo. Census of Population and Housing: Estimating Homelessness. Canberra, ACT: Statistics ABo (2018)

[2] McVicar D Moschion J van Ours JC. From substance use to homelessness or vice versa? Soc Sci Med. (2015) 136-137:89-98. 10.1016/j.socscimed.2015.05.005 25989002

[3] Stablein GW Hill BS Keshavarz S Llorente MD. Homelessness and substance use disorders. In: Ritchie EC Llorente MD editors. Clinical Management of the Homeless Patient: Social, Psychiatric, and Medical Issues. Cham: Springer International Publishing (2021). p. 179-94.

[4] Moxley VB Hoj TH Novilla MLB. Predicting homelessness among individuals diagnosed with substance use disorders using local treatment records. Addict Behav. (2020) 102:106160. 10.1016/j.addbeh.2019.106160 31841870

[5] Upshur CC Jenkins D Weinreb L Gelberg L Orvek EA. Homeless women's service use, barriers, and motivation for participating in substance use treatment. Am J Drug Alcohol Abuse. (2018) 44:252-62. 10.1080/00952990.2017.1357183 28806101 PMC6088786

[6] Health AIo Welfare. Specialist Homelessness Services Annual Report. Canberra: AIHW (2020).

[7] Russell C Ali F Nafeh F LeBlanc S Imtiaz S Elton-Marshall T. A qualitative examination of substance use service needs among people who use drugs (PWUD) with treatment and service experience in Ontario, Canada. BMC Public Health. (2021) 21:2021. 10.1186/s12889-021-12104-w 34742267 PMC8571863

[8] Omerov P Craftman ÅG Mattsson E Klarare A. Homeless persons' experiences of health- and social care: a systematic integrative review. Health Soc Care Commun. (2020) 28:1-11. 10.1111/hsc.12857 31524327

[9] Davies A Wood LJ. Homeless health care: meeting the challenges of providing primary care. Med J Aust. (2018) 209:230-4. 10.5694/mja17.01264 30157413

[10] Johnson G Scutella R Tseng Y-P Wood G. Examining the Relationship Between Structural Factors, Individual Characteristics, and Homelessness. Melbourne: Australian Housing and Urban Research Institute Limited (2015). Report No.: AHURI Positioning Paper No. 161.

[11] Chamberlain C Johnson G Robinson C Chamberlain C Johnson G Robinson C. Homelessness in Australia. Sydney, NSW: UNSW Press (2014).

[12] Tessler R Rosenheck R Gamache G. Gender differences in self-reported reasons for homelessness. J Soc Distress Homeless. (2001) 10:243-54. 10.1023/A:1016688707698

[13] Winetrobe H Wenzel S Rhoades H Henwood B Rice E Harris T. Differences in health and social support between homeless men and women entering permanent supportive housing. Womens Health Issues. (2017) 27:286-93. 10.1016/j.whi.2016.12.011 28153741 PMC5435523

[14] Anderson DG Rayens MK. Factors influencing homelessness in women. Public Health Nurs. (2004) 21:12-23. 10.1111/j.1525-1446.2004.21103.x 14692985

[15] Beijer U Andréasson S. Physical diseases among homeless people: gender differences and comparisons with the general population. Scand J Public Health. (2009) 37:93-100. 10.1177/1403494808099972 19141558

[16] Aldridge RW Story A Hwang SW Nordentoft M Luchenski SA Hartwell G. Morbidity and mortality in homeless individuals, prisoners, sex workers, and individuals with substance use disorders in high-income countries: a systematic review and meta-analysis. Lancet. (2018) 391:241-50. 10.1016/S0140-6736(17)31869-X 29137869 PMC5803132

[17] Duke A Searby A. Mental ill health in homeless women: a review. Issues Ment Health Nurs. (2019) 40:605-12. 10.1080/01612840.2019.1565875 31021673

[18] Belcher JR Greene JA McAlpine C Ball K. Considering pathways into homelessness: mothers, addictions, and trauma. J Addict Nurs. (2001) 13:199-208. 10.3109/10884600109052654

[19] Phipps M Dalton L Maxwell H Cleary M. Women and homelessness, a complex multidimensional issue: findings from a scoping review. J Soc Distress Homelessness. (2019) 28:1-13. 10.1080/10530789.2018.1534427

[20] Rodriguez-Moreno S Vázquez JJ Roca P Panadero S. Differences in stressful life events between men and women experiencing homelessness. J Community Psychol. (2021) 49:375-89. 10.1002/jcop.22465 33131105

[21] Song A Wenzel SL Cho Y. Child abuse victimization, depression, and substance use among homeless women: application of general strain theory. J Interpers Violence. (2019) 36:8852-73. 10.1177/0886260519853410 31179812

[22] Broll R Huey L. “Every time I try to get out, I get pushed back”: the role of violent victimization in women's experience of multiple episodes of homelessness. J interpers Violence. (2020) 35:3379-404. 10.1177/0886260517708405 29294758

[23] Bonugli R Lesser J Escandon S. “The second thing to hell is living under that bridge”: narratives of women living with victimization, serious mental illness, and in homelessness. Issues Ment Health Nurs. (2013) 34:827-35. 10.3109/01612840.2013.831149 24131415

[24] Cronley C Hohn K Nahar S. Reproductive health rights and survival: the voices of mothers experiencing homelessness. Women Health. (2018) 58:320-33. 10.1080/03630242.2017.1296060 28278012

[25] Gelberg L Browner CH Lejano E Arangua L. Access to women's health care: a qualitative study of barriers perceived by homeless women. Women Health. (2004) 40:87-100. 10.1300/J013v40n02_06 15778140

[26] Azim KA MacGillivray L Heise D. Mothering in the margin: a narrative inquiry of women with children in a homeless shelter. J Soc Distress Homelessness. (2019) 28:34-43. 10.1080/10530789.2018.1548091

[27] Becker JB McClellan ML Reed BG. Sex differences, gender and addiction. J Neurosci Res. (2017) 95:136-47. 10.1002/jnr.23963 27870394 PMC5120656

[28] Sanchis-Segura C Becker JB. Why we should consider sex (and study sex differences) in addiction research. Addict Biol. (2016) 21:995-1006. 10.1111/adb.12382 27029841 PMC5585537

[29] McHugh RK Votaw VR Sugarman DE Greenfield SF. Sex and gender differences in substance use disorders. Clin Psychol Rev. (2018) 66:12-23. 10.1016/j.cpr.2017.10.012 29174306 PMC5945349

[30] Becker JB McClellan M Reed BG. Sociocultural context for sex differences in addiction. Addict Biol. (2016) 21:1052-9. 10.1111/adb.12383 26935336 PMC5555215

[31] Erol A Ho AMC Winham SJ Karpyak VM. Sex hormones in alcohol consumption: a systematic review of evidence. Addict Biol. (2019) 24:157-69. 10.1111/adb.12589 29280252 PMC6585852

[32] Peltier MR Sofuoglu M. Role of exogenous progesterone in the treatment of men and women with substance use disorders: a narrative review. CNS Drugs. (2018) 32:421-35. 10.1007/s40263-018-0525-5 29761343 PMC6235727

[33] Valeri L Sugarman DE Reilly ME McHugh RK Fitzmaurice GM Greenfield SF. Group therapy for women with substance use disorders: In-session affiliation predicts women's substance use treatment outcomes. J Subst Abuse Treat. (2018) 94:60-8. 10.1016/j.jsat.2018.08.008 30243419 PMC9976621

[34] Holzhauer CG Cucciare M Epstein EE. Sex and gender effects in recovery from alcohol use disorder. Alcohol Res Curr Rev. (2019) 40:1-19. 10.35946/arcr.v40.3 33224697 PMC7668196

[35] Barnett ER Knight E Herman RJ Amarakaran K Jankowski MK. Difficult binds: a systematic review of facilitators and barriers to treatment among mothers with substance use disorders. J Subst Abuse Treat. (2021) 126:108341. 10.1016/j.jsat.2021.108341 34116826

[36] Brogly SB Link K Newman A for the Kingston House of Recovery for W Children. Barriers to treatment for substance use disorders among women with children. Can J Addiction. (2018) 9:18-22. 10.1097/CXA.0000000000000025

[37] Frazer Z McConnell K Jansson LM. Treatment for substance use disorders in pregnant women: motivators and barriers. Drug Alcohol Depend. (2019) 205:107652. 10.1016/j.drugalcdep.2019.107652 31704383

[38] Stone R. Pregnant women and substance use: fear, stigma, and barriers to care. Health Justice. (2015) 3:2. 10.1186/s40352-015-0015-5

[39] Agterberg S Schubert N Overington L Corace K. Treatment barriers among individuals with co-occurring substance use and mental health problems: examining gender differences. J Subst Abuse Treat. (2020) 112:29-35. 10.1016/j.jsat.2020.01.005 32199543

[40] Batchelder AW Stanton AM Kirakosian N King D Grasso C Potter J. Mental health and substance use diagnoses and treatment disparities by sexual orientation and gender in a community health center sample. LGBT Health. (2021) 8:290-9. 10.1089/lgbt.2020.0293 34080895 PMC8213009

[41] Brand E Rodriguez-Monguio R Volberg R. Gender differences in mental health and substance use disorders and related healthcare services utilization. Am J Addictions. (2019) 28:9-15. 10.1111/ajad.12826 30536669

[42] Priester MA Browne T Iachini A Clone S DeHart D Seay KD. Treatment access barriers and disparities among individuals with co-occurring mental health and substance use disorders: an integrative literature review. J Subst Abuse Treat. (2016) 61:47-59. 10.1016/j.jsat.2015.09.006 26531892 PMC4695242

[43] Health AIo Welfare. Alcohol and other drug treatment services in Australia: early insights. Canberra: AIHW (2021).

[44] Callander EJ Fox H Lindsay D. Out-of-pocket healthcare expenditure in Australia: trends, inequalities and the impact on household living standards in a high-income country with a universal health care system. Health Econ Rev. (2019) 9:10. 10.1186/s13561-019-0227-9 30859357 PMC6734455

[45] Tucker JS Wenzel SL Golinelli D Zhou A Green HD. Predictors of substance abuse treatment need and receipt among homeless women. J Subst Abuse Treat. (2011) 40:287-94. 10.1016/j.jsat.2010.11.006 21185682 PMC3056903

[46] Kneck Å Mattsson E Salzmann-Erikson M Klarare A. “Stripped of dignity” – women in homelessness and their perspectives of healthcare services: a qualitative study. Int J Nurs Stud. (2021) 120:103974. 10.1016/j.ijnurstu.2021.103974 34087526

[47] Gale NK Heath G Cameron E Rashid S Redwood S. Using the framework method for the analysis of qualitative data in multi-disciplinary health research. BMC Med Res Methodol. (2013) 13:117. 10.1186/1471-2288-13-117 24047204 PMC3848812

[48] Berger MT Berger MTT. Workable Sisterhood : The Political Journey of Stigmatized Women with HIV/AIDS. Princeton, NJ: Princeton University Press (2006).

[49] Chambers BD Erausquin JT. The promise of intersectional stigma to understand the complexities of adolescent pregnancy and motherhood. J Child Adolesc Behav. (2015) 3:249. 10.4172/2375-4494.1000249

[50] Thomas N Menih H. Negotiating multiple stigmas: substance use in the lives of women experiencing homelessness. Int J Mental Health Addict. (2021). 10.1007/s11469-021-00560-9. [Epub ahead of print].

[51] Margaretha Strandmark K. Ill health is powerlessness: a phenomenological study about worthlessness, limitations and suffering. Scand J Caring Sci. (2004) 18:135-44. 10.1111/j.1471-6712.2004.00275.x 15147476

[52] Van Den Tillaart S Kurtz D Cash P. Powerlessness, marginalized identity, and silencing of health concerns: Voiced realities of women living with a mental health diagnosis. Int J Ment Health Nurs. (2009) 18:153-63. 10.1111/j.1447-0349.2009.00599.x 19490225

[53] Yang C Zhou Y Cao Q Xia M An J. The relationship between self-control and self-efficacy among patients with substance use disorders: resilience and self-esteem as mediators. Front Psychiatry. (2019) 10:388. 10.3389/fpsyt.2019.00388 31249535 PMC6582546

[54] Hyde J Hankins M Deale A Marteau TM. Interventions to increase self-efficacy in the context of addiction behaviours: a systematic literature review. J Health Psychol. (2008) 13:607-23. 10.1177/1359105308090933 18519435

[55] Kadden RM Litt MD. The role of self-efficacy in the treatment of substance use disorders. Addict Behav. (2011) 36:1120-6. 10.1016/j.addbeh.2011.07.032 21849232 PMC3179802

[56] Carver H Ring N Miler J Parkes T. What constitutes effective problematic substance use treatment from the perspective of people who are homeless? A systematic review and meta-ethnography. Harm Reduct J. (2020) 17:10. 10.1186/s12954-020-0356-9 32005119 PMC6995160

[57] Elms N Link K Newman A Brogly SB. Need for women-centered treatment for substance use disorders: results from focus group discussions. Harm Reduct J. (2018) 15:1-8. 10.1186/s12954-018-0247-5 30081905 PMC6080513

[58] Gressler LE Natafgi NM DeForge B Shaneman-Robinson B Welsh C Shaya F. What motivates people with substance use disorders to pursue treatment? A patient-centered approach to understanding patient experiences and patient-provider interactions. J Substance Use. (2019) 24:587-99. 10.1080/14659891.2019.1620891 31582894 PMC6776437

